# Millimeter-Level MEMS Actuators Based on Multi-Folded Beams and Harmful Mode-Suppression Structures

**DOI:** 10.3390/mi17010144

**Published:** 2026-01-22

**Authors:** Hangyu Zhou, Wei Bian, Rui You

**Affiliations:** 1 School of Instrument Science and Optoelectronics Engineering, Beijing Information Science & Technology University, Beijing 100192, China; zhouhy68@foxmail.com; 2 Department of Precision Instrument, Tsinghua University, Beijing 100084, China; bianw11@foxmail.com

**Keywords:** MEMS actuator, Differential Motion Rejection, folded beam, out-of-plane stiffness, module-level optical interconnect

## Abstract

Module-level free-space optical interconnects require actuators to combine both large stroke and high stability. To address this core trade-off that plagues traditional folded-beam actuators, we have developed a millimeter-scale MEMS electromagnetic actuator integrating a Differential Motion Rejection (DMR) unit with a rigid frame. Its performance was systematically evaluated through magnetic–structural coupling modeling, finite element simulation, and experiments. The actuator achieved millimeter-scale stroke under sinusoidal drive, with a primary resonant frequency of approximately 31 Hz. The introduction of the DMR and frame proved highly effective: the out-of-plane displacement at resonance was reduced by about 97%, the static Z-direction stiffness increased by over 50 times, and the displacement crosstalk decreased to 0.265%. Optical testing yielded a stable deflection angle of approximately ±21°. These results demonstrate that this design successfully combines large stroke with high stability, significantly suppressing out-of-plane parasitic motion and crosstalk, making it suitable for module-level optical interconnect systems with stringent space and stability requirements.

## 1. Introduction

Optical interconnect technology is extending closer to devices at the module level to meet the continuously growing demands for bandwidth and energy efficiency [1,2]. Module-level free-space optical interconnection redirects optical paths through free-space propagation within boards, allowing flexible connection adjustments at millimeter and centimeter scales. This approach reduces the need for fiber patch cords and complex couplers, thereby lowering the packaging volume and alignment complexity.

Currently, two main technical paths exist for achieving beam steering. One is the rotating micromirror matrix scheme, which is sufficient for small-angle applications but faces significant bottlenecks when pursuing large deflection angles [3]. Research by Torres et al. on MEMS micromirrors indicates that achieving beam deflection angles beyond 20° remains a challenging technical problem [4]. Hinges are prone to out-of-plane deformation and nonlinear coupling under large-angle operation, leading to reduced optical alignment accuracy. Moreover, achieving larger deflection angles often requires designing more complex hinge structures or multi-stage transmission mechanisms, which increases device fabrication and packaging costs while raising control complexity [5]. The other path involves adjusting the optical path by translating lenses or light sources, where the mapping between displacement and angle is more direct, and linear displacement can be amplified into larger optical deflection angles [6,7]. However, the translational approach faces considerable challenges in ensuring high alignment stability under large strokes. The difficulty lies in the fact that folded beams providing large strokes must possess extremely high out-of-plane stiffness to suppress parasitic motion, and large displacements amplify the effects of manufacturing and assembly tolerances. Therefore, controlling out-of-plane displacement and cross-axis crosstalk and ensuring assembly consistency are core to the engineering application of translational schemes [8,9,10]. Mohamed et al. proposed a beam-steering technique based on different optical lenses, theoretically enhancing and directing light radiation in specific directions by optimizing the illuminator element configuration [11].

In addition, electrothermally driven multibeam/bimorph actuators have made progress in the field of “large displacement + low parasitic motion”. Wu et al. [12] proposed a three-bimorph electrothermal actuator, achieving a vertical displacement of 0.62 mm and a low lateral shift of 10 μm with a driving voltage of only 5.3 V; Yang et al. [13] significantly improved device impact resistance by using photosensitive polyimide (PSPI) instead of ${\mathrm{SiO}}_{2}$ as the anchoring material, while obtaining a displacement of 370 μm and an optical scan angle of ±19.6°. However, such electrothermally driven designs have inherent shortcomings: the response time is as long as 25~70 ms, and the displacement is mostly limited to hundreds of micrometers, which is difficult to meet the requirements of module-level optical interconnects for millimeter-scale stroke and fast switching. Therefore, this study adopts a synergistic design of electromagnetic drive combined with a DMR unit and a rigid frame. While avoiding the limitations of electrothermal drive, it achieves millimeter-scale large stroke, 54-fold stiffness enhancement, and 34-fold crosstalk optimization, making it more suitable for harsh application scenarios.

Based on the aforementioned requirements, this work designs a millimeter-stroke MEMS electromagnetic actuator. The design is centered on using multiple folded beams to support a micro-lens-integrated mass block, thereby directly mapping mechanical translation to optical deflection. To address the parasitic motion problem under large strokes, a Differential Motion Rejection (DMR) unit and an external rigid frame are introduced into the structure. Their combination effectively enhances the actuator’s out-of-plane stiffness and suppresses cross-axis crosstalk [14]. Key experimental data from the prototype are as follows: drive voltage range ±5 V, dynamic maximum stroke 674 μm, and primary resonant frequency approximately 31 Hz. After introducing DMR and the rigid frame, the out-of-plane displacement near resonance significantly decreased from about 57.6 μm to about 1.7 μm, static Z-direction stiffness increased from 9.62 N/m to 519.3 N/m, displacement crosstalk decreased from about 8.97% to 0.265%, and optical deflection was about ±21°. These results demonstrate a significant improvement in out-of-plane stability and crosstalk control while maintaining large displacement, consequently enhancing optical alignment quality.

The characteristics of this actuator make it particularly suitable for module-level reconfigurable optical interconnect applications. Such applications require reliable optical path redirection and stable coupling within compact spaces. The actuator’s high out-of-plane stiffness and low crosstalk help maintain alignment accuracy post-packaging, while its large optical deflection angle has the potential to reduce relay stages in the system, thereby lowering overall packaging complexity and alignment costs. The primary resonant frequency is 31 Hz, indicating that for applications not requiring extremely fast switching, reliable optical path reconfiguration operations can be achieved by optimizing the drive scheme. Subsequent sections will detail the mechanical simulation process, frequency-response testing methods, quantitative displacement–angle mapping relationship, and preliminary system-level evaluation results of this actuator in module-level interconnects. Notably, the core innovation of this work is the integration of the DMR unit and rigid frame into the folded-beam structure, forming a synergistic constraint system that enhances out-of-plane stiffness without sacrificing in-plane stroke—this is the first attempt to address the “large stroke vs. high stability” dilemma through such a three-layer composite structure in module-level MEMS actuators.

## 2. Materials and Methods

### 2.1. Magnetic Field Crosstalk Modeling and Cross-Axis Crosstalk

The actuator is an overall square structure with a side length of 13,000 μm and a thickness of 300 μm. Its electromagnetic drive system consists of a composite structure of a central mass block and a circular permanent magnet, as well as a planar coil. The central mass block adopts a square silicon-based design with dimensions of 5800 μm × 5800 μm × 300 μm. A circular neodymium–iron–boron permanent magnet (grade N52, diameter 6 mm, thickness 1 mm, saturation magnetization $M_{s}=1.48T$, residual flux density $B_{r}=1.32T$, permeability $\mu_{r}=1.05$) is bonded and fixed to the lower surface of the mass block using high-temperature-resistant epoxy adhesive. The bonding layer has a thickness of 10 μm.

The driving coil matched with the permanent magnet is a five-turn planar spiral structure, with a line width of 50 μm, a line spacing of 30 μm, a copper wire thickness of 10 μm, and an effective side length of 6000 μm. The effective side length of the coil is consistent with the diameter of the permanent magnet, which ensures that the magnetic field can fully cover the permanent magnet, thereby maximizing the X-direction driving force and achieving precise electromagnetic coupling between the two.

**Figure 1:**
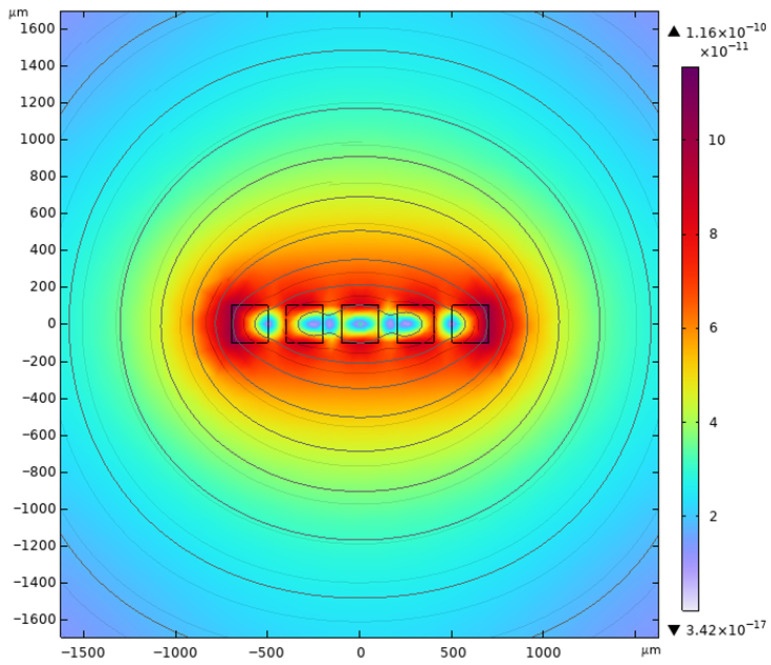
Coil magnetic field distribution.

To quantify cross-axis (Z-direction) coupling induced by the driving coil, the magnetic field at the actuator’s working plane must be modeled. Finite-element simulations are conducted for a coil model composed of five equal-spaced wires with an effective side length of 200 μm; the resulting magnetic field distribution is shown in Figure 1.

The magnetic field analysis plane was set 2 mm above the coil to match the actual assembly gap. On this plane, the magnetic field was sampled over a 200 μm wide region. The magnetic field gradient at this location is moderate, which is beneficial for obtaining sufficient driving force while controlling cross-axis coupling strength.

Based on the simulation data, the magnitude of the magnetic flux density B(x,z) is fitted with a second-order polynomial of x and z:

(1) $$B(x,z)\approx c_{0}+c_{1}x+c_{2}z+c_{3}x^{2}+c_{4}xz+c_{5}z^{2}$$

The fitted coefficients are as follows:

(2) $$\begin{array}{l} c_{0}=4.98\times 10^{-11}\\ c_{1}=3.14\times 10^{-17}\\ c_{2}=1.70\times 10^{-17}\\ c_{3}=-5.26\times 10^{-18}\\ c_{4}=1.00\times 10^{-20}\\ c_{5}=-5.96\times 10^{-18} \end{array}$$

Under large displacements, the non-uniform magnetic field induces electromagnetic coupling, resulting in additional Z-direction displacement. To evaluate this effect, the displacement crosstalk (cross-axis ratio) is defined as the ratio of the maximum Z-direction displacement to the maximum X-direction displacement:

(3) $$\chi =\frac{\Delta z_{\max}}{\Delta x_{\max}}$$

This ratio directly reflects the severity of out-of-plane displacement relative to the working stroke and provides an intuitive engineering metric for actuator performance evaluation.

### 2.2. Structural Stiffness Modeling

The structural stiffness modeling of the actuator is based on the Euler–Bernoulli beam theory (assuming cross-sections remain planar during bending and neglecting shear deformation). Verification on the slimmest beam in the design (L = 6000 μm, W = 20 μm, T = 300 μm) shows that the shear deformation ratio is only 0.8%, well below the 5% engineering threshold, confirming the applicability of this theory.

**Figure 2:**
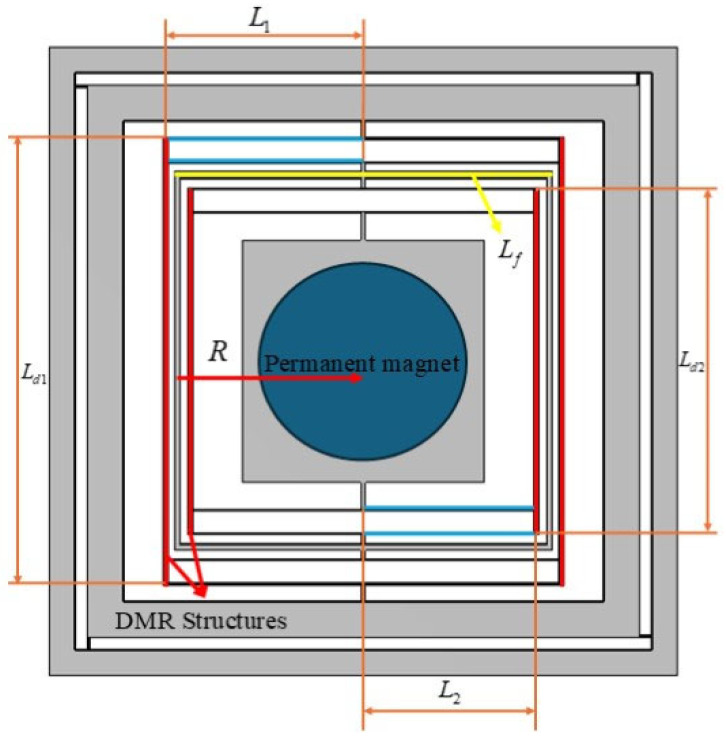
Structural design diagram of electromagnetic actuator.

As shown in Figure 2, the actuator adopts a three-layer composite structure, and its out-of-plane (Z-direction) stiffness is synergistically contributed by the basic stiffness of folded beams ( $k_{Z,0}$), differential suppression stiffness of DMR beams ( $k_{Z,DMR}$), and boundary enhancement stiffness of the square frame ( $k_{Z,frame}$). The total stiffness expression is given by

(4) $${k_{Z}}^{total}=k_{Z,DMR}+\frac{k_{Z,0}k_{Z,frame}}{k_{Z,0}+k_{Z,frame}}$$

The core innovation of this design lies in the synergistic effect of the three—the DMR beam enhances the dominant out-of-plane stiffness, the frame supplements boundary stability, and the folding beam balances the in-plane large stroke and foundation out-of-plane stiffness, ultimately overcoming the challenge faced by traditional folding-beam actuators that cannot achieve both large stroke and high stability. The stiffness contribution of each part is derived as follows.

#### 2.2.1. Stiffness Contribution of Folded Beams

The folded beams serve as the core support for the large X-direction stroke, with its Z-direction bending stiffness derived from geometric parameters (segment lengths $L_{1}$, $L_{2}$; width W; thickness T). Each set of folded beams consists of cantilever segments connected in series, and three such sets are arranged in parallel. Combined with the symmetric upper and lower folded beams connected to the mass block, the equivalent Z-direction stiffness is

(5) $$k_{Z,0}=\frac{EWT^{3}}{2}(\frac{2}{{L_{1}}^{3}}+\frac{2}{{L_{2}}^{3}}+\frac{1}{{L_{1}}^{3}+{L_{2}}^{3}})$$

The material is isotropic silicon. Stress concentrations, thermal residual stresses, and contact nonlinearities are neglected. Beams operate within the small-deformation linear regime. Parameters are listed in Table 1.

#### 2.2.2. Stiffness Contribution of DMR Beams

Two groups of DMR beams with different lengths ( $L_{d1}$, $L_{d2}$) are symmetrically arranged to suppress Z-direction differential warping. When the folded beams undergo differential out-of-plane displacement, the DMR beams act as fixed–fixed beams under symmetric loads, and their equivalent stiffness is the parallel combination of four beams (two for each length):

(6) $$k_{z,DMR}=2k_{d1}+2k_{d2}=2\cdot \frac{16EW_{d}T^{3}}{L_{d1}^{3}}+2\cdot \frac{16EW_{d}T^{3}}{L_{d2}^{3}}=32EW_{d}T^{3}(\frac{1}{L_{d1}^{3}}+\frac{1}{L_{d2}^{3}})$$

where $k_{d1}$ and $k_{d2}$ are the stiffness of DMR beams with lengths $L_{d1}$ and $L_{d2}$, respectively.

#### 2.2.3. Stiffness Contribution of the Square Frame

The closed square frame is embedded between the inner and outer DMR beams, which does not directly bear the Z-direction load of the mass block but restricts the overall torsion of the DMR structure through bending deformation. The equivalent Z-direction stiffness contributed by the four edge beams of the frame is

(7) $$k_{z,frame}=\frac{64EW_{f}T^{3}R}{L_{f}^{3}}$$

A key innovation of this design lies in the synergistic effect of the three stiffness components: the DMR beams dominate the out-of-plane stiffness enhancement, while the frame supplements boundary stability and the folded beams balance in-plane stroke and basic out-of-plane stiffness. This collaborative design achieves a 54-fold increase in Z-direction stiffness compared to the structure without a DMR and frame, resolving the trade-off between large stroke and high stability that plagues traditional folded-beam actuators.

### 2.3. Actuator Mechanical Simulation

**Figure 3:**
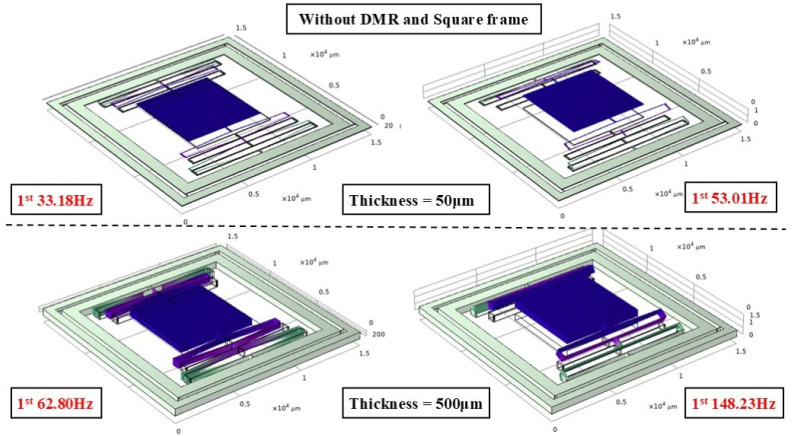
Modal simulation of structure without DMR structure.

**Figure 4:**
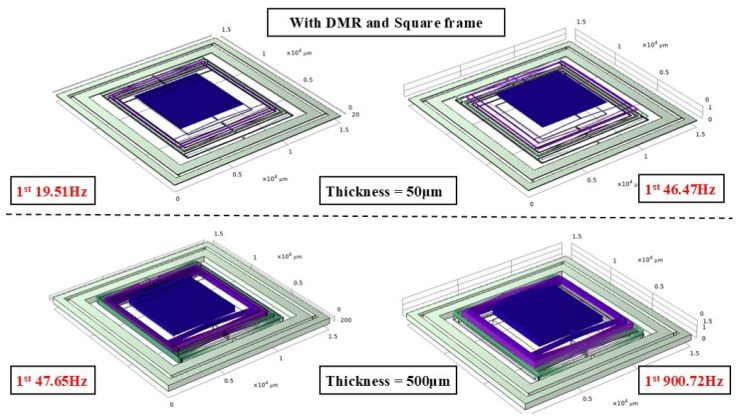
Modal simulation of structure with DMR structure.

**Figure 5:**
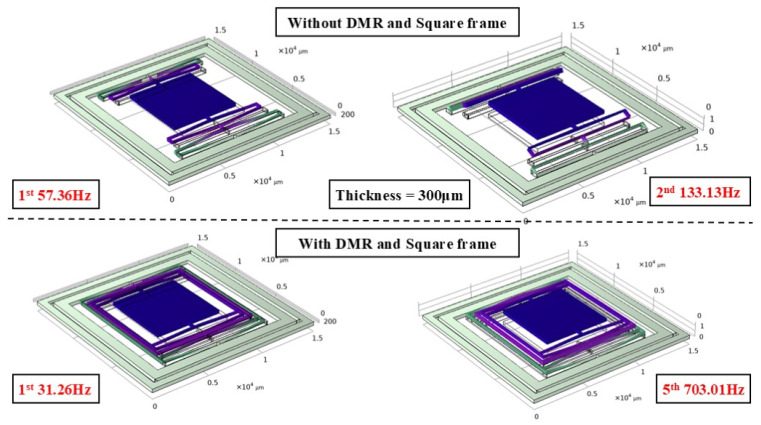
300-micron actuator modal simulation.

**Figure 6:**
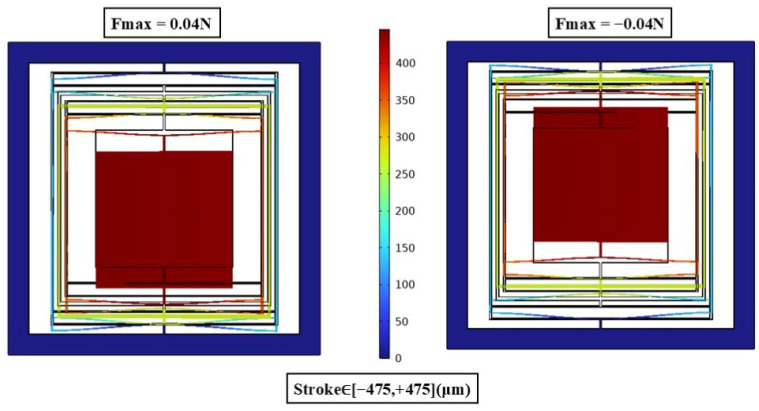
Actuator travel simulation.

To verify the impact of the DMR structure on the modal characteristics, finite element analysis was conducted, focusing on examining the changes in modal frequencies of the actuator with and without the DMR structure, particularly at different thicknesses. Figure 3, Figure 4 and Figure 5 show the mode-shape comparisons for the two structures, respectively. Furthermore, the designed maximum stroke of the actuator was simulated, as shown in Figure 6.

It should be noted that the resonant frequency is positively correlated with the out-of-plane (Z-direction) stiffness. Therefore, the comparison of modal frequencies between the two configurations (with and without DMR and frame) can intuitively highlight the improvement effect of out-of-plane stiffness. This simulation comparison mutually corroborates with the measured stiffness data in Section 3.4 of the experiment. Specifically:

For the 50 μm thick structure, the out-of-plane modal frequency of the base configuration (without DMR and frame) is 186.16 Hz, which increases to 837.22 Hz (approximately 4.5 times higher) after integrating the DMR and frame; for the 300 μm thick structure, the out-of-plane modal frequency of the base configuration is 133.13 Hz, while that of the coupled configuration (with DMR and frame) jumps to 703.01 Hz (approximately 5.3 times higher); for the 500 μm thick structure, the out-of-plane modal frequency rises from 184.55 Hz to 1644.16 Hz (nearly 9 times higher). This indicates that the DMR structure can selectively and significantly enhance Z-direction stiffness, effectively suppressing out-of-plane motion without affecting in-plane performance. The designed stroke of this actuator is 950 μm.

## 3. Results

**Figure 7:**
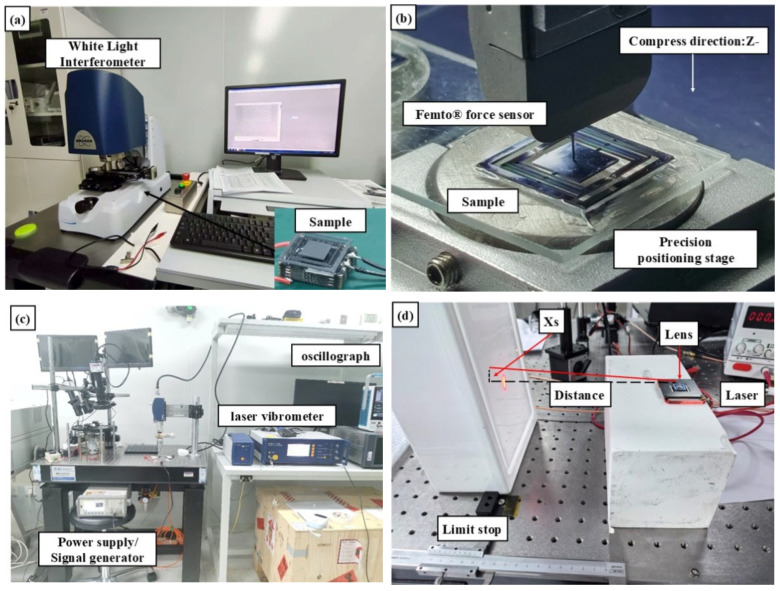
Experimental photos. Designed the environment for in-plane stroke testing (**a**) and stiffness testing (**b**) of MEMS drivers; environmental settings for out-of-plane travel test (**c**) and light deflection angle test (**d**).

The aforementioned theory and design were verified through experiments, including the static voltage-displacement characteristics, frequency-response, out-of-plane vibration comparison, Z-direction static stiffness measurement, actuator optical deflection angle, and calculation of crosstalk based on experimental data. An overview of the experimental setup is shown in Figure 7.

### 3.1. Static Voltage-Driven X-Direction Displacement

**Figure 8:**
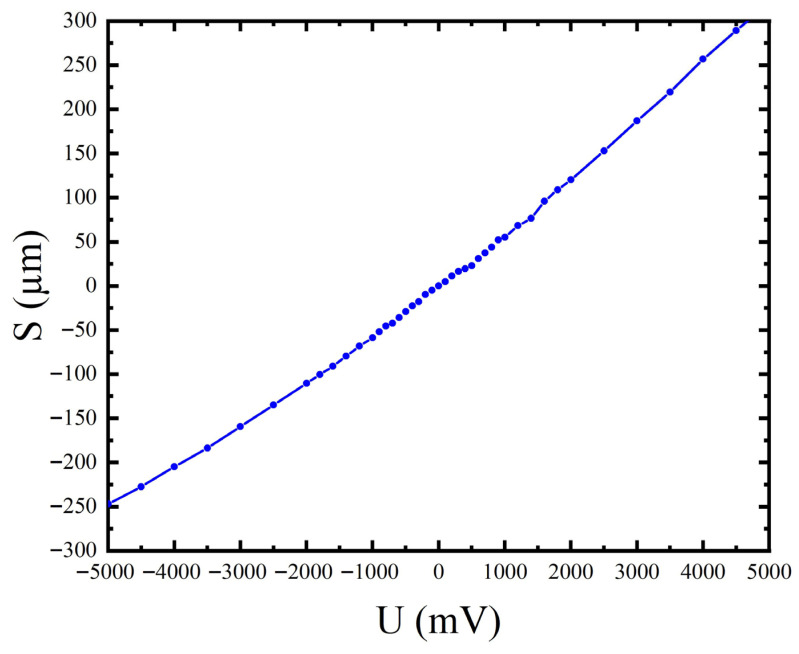
Static voltage and X-direction displacement curve.

The constant voltage source is used to drive the X-direction coil, and the white light interferometer is used to measure the displacement. The voltage is scanned from −5 V to +5 V, and multiple frames of data are collected at each steady-state point to take the mean value. The results are shown in Figure 8.

The voltage–displacement curve shows good linearity and symmetry within the test range. The typical data points are at +5.5 V and −5.5 V, the displacement reaches +474.5 μm and −476.1 μm, respectively. When the voltage rises to 6 V, the displacement saturation occurs. Static sensitivity is calculated based on ±5.5 V data

(8) $$S=\frac{474.5-(-476.125)}{5500-(-5500)}\approx 0.0864\;\mu m/\mathrm{mV}$$

Thus, in the non-saturated region, Δx≈S⋅V.

### 3.2. Frequency-Response Test and Resonant Frequency Determination

**Figure 9:**
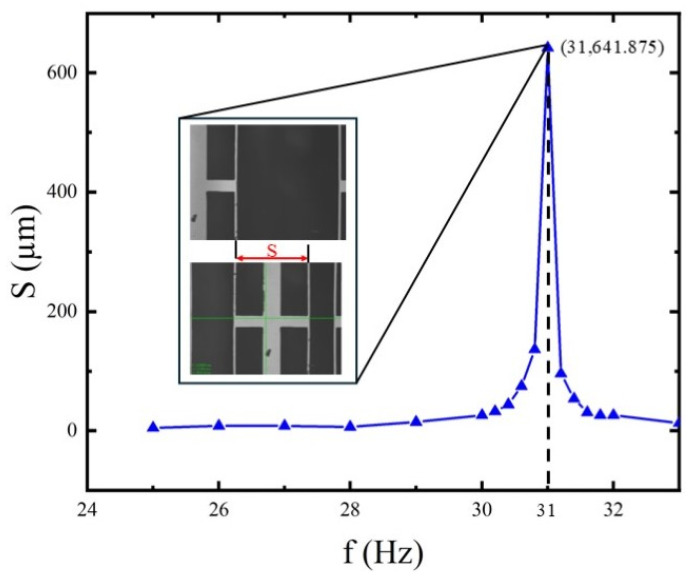
Amplitude–frequency response curve of actuator.

The coil was driven by a sinusoidal voltage with amplitude 30 mV into a 50 Ω load. Frequency sweeping used a coarse-to-fine approach: a coarse sweep with 1 Hz steps over 25–30 Hz, then a fine sweep of 0.2 Hz steps near the peak (30.2–32 Hz), and additional points at 33 and 34 Hz to observe the decay. At each frequency, the mean X-amplitude was recorded; the amplitude–frequency curve is shown in Figure 9.

The response curve shows obvious unimodal characteristics. The resonance frequency is $f_{0}=30\;\mathrm{Hz}$, and the maximum displacement is $\Delta x_{\max}=641.875\;\mu m$.

### 3.3. Out-of-Plane Vibration Comparison: Base vs. Coupled-Stiffness Configurations

**Figure 10:**
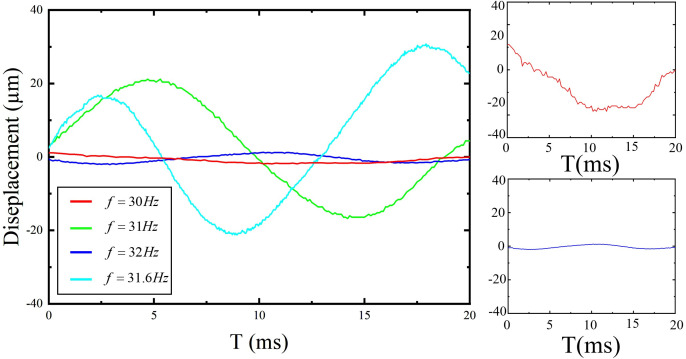
Vibration waveform of foundation configuration.

**Figure 11:**
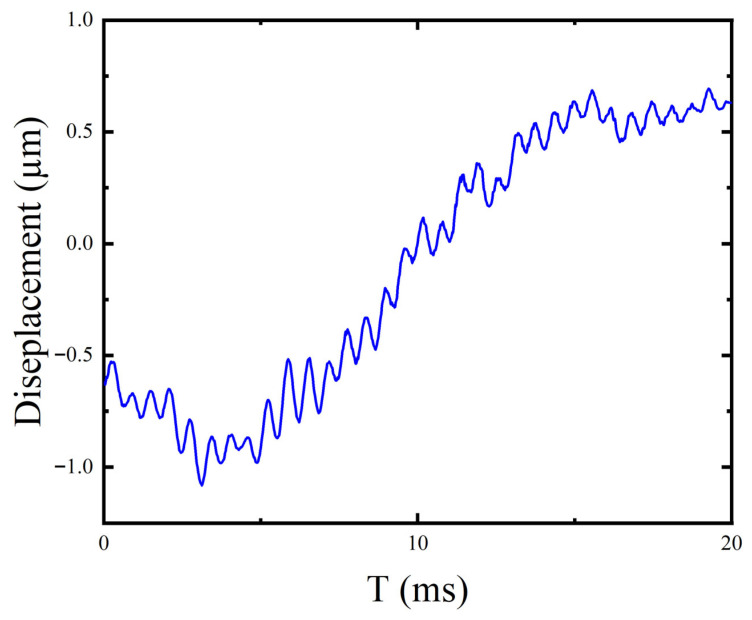
Vibration waveform of coupled-stiffness configuration.

This experiment compares out-of-plane vibration responses of the base configuration (without DMR and frame) and the coupled-stiffness configuration (with DMR and frame). Waveforms were recorded by a laser vibrometer; results are shown in Figure 10 and Figure 11.

**Figure 12:**
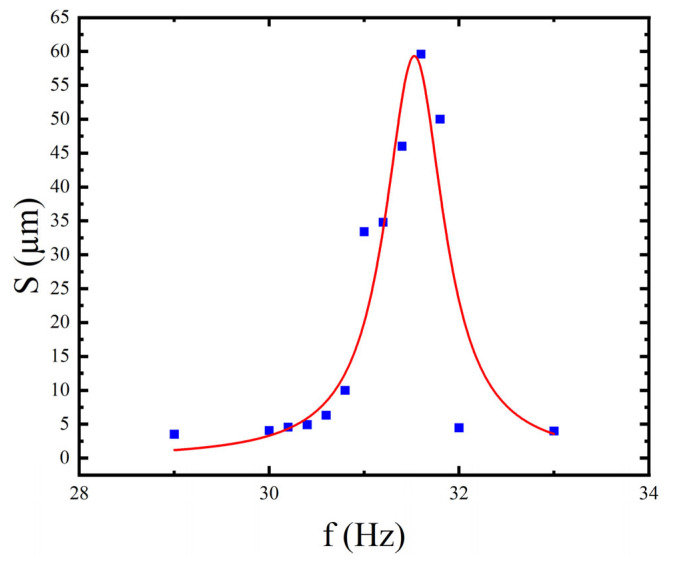
Z-axis out-of-plane motion stroke and input frequency test results.

For the base configuration, the peak out-of-plane displacement reaches 57.6 μm at 31.6 Hz. In contrast, the displacement near 31 Hz for the coupled-stiffness configuration is significantly suppressed, reaching only 1.7 μm. This directly confirms the effectiveness of the DMR structure in suppressing Z-direction vibration. To extract characteristic parameters, we employed a Lorentzian function model to perform nonlinear least-squares fitting on the Z-direction frequency-response data. The function form is

(9) $$S(f)=S_{\max}\frac{{\left(FWHM/2\right)}^{2}}{{\left(f-f_{C}\right)}^{2}+{\left(FWHM/2\right)}^{2}}$$

where $S_{\max}$ is peak displacement, $f_{c}$ is center frequency, and FWHM is full width at half maximum. Fit results are in Figure 12; the model extracts key response parameters and confidence intervals, further quantifying the DMR’s suppression effect.

### 3.4. Z-Direction Static Stiffness Measurement

**Figure 13:**
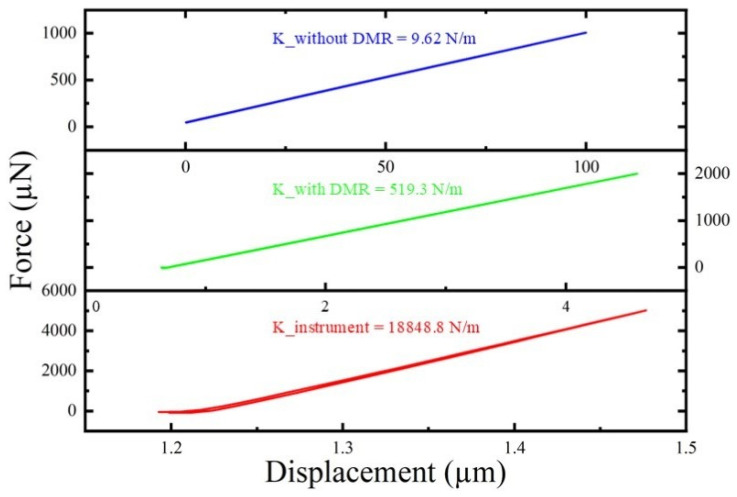
Actuator stiffness measurement curve.

A micro-/nano-force sensor applied to static loads up to 200 μN at the platform center while Z displacements Δz were recorded. Measurements were taken first on the coupled-stiffness configuration and, then, on the base configuration after removing the DMR and frame. Linear fits of the force–displacement curves yield stiffness: $k_{z}=\frac{dF}{d\Delta z}$, as shown in Figure 13.

The fitting results are as follows: instrument body stiffness $k_{plat}=18,848.8\;N/m$, coupling stiffness configuration $k_{couple}=519.3\;N/m$, and foundation configuration $k_{base}=9.62\;N/m$. The Z-direction stiffness of the coupled-stiffness configuration is about 54 times higher than that of the basic configuration.

Displacement crosstalk for the base and coupled configurations are

(10) $$\left\{\begin{array}{l} \chi_{base}=\frac{57.6}{641.875}\approx 8.97\times 10^{-2}\\ \chi_{couple}=\frac{1.7}{641.875}\approx 2.65\times 10^{-3} \end{array}\right.$$

Introducing the coupled-stiffness configuration reduces crosstalk from approximately $8.97\times 10^{-2}$ to $2.65\times 10^{-3}$, an improvement of roughly 34×. This demonstrates the DMR and rectangular frame’s effectiveness in suppressing out-of-plane displacement and reducing cross-axis interference.

### 3.5. Light Deflection Angle Experiment

**Figure 14:**
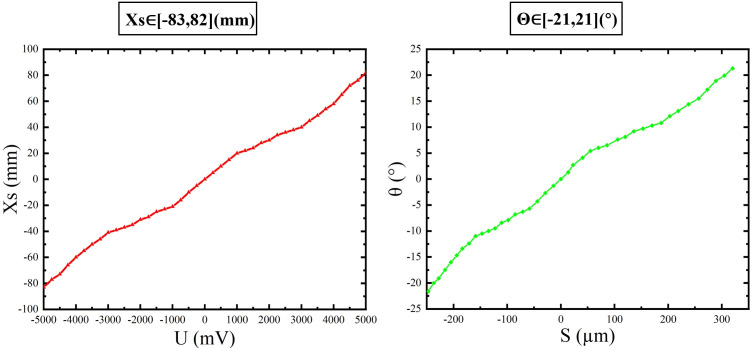
Optical angle measurement experimental diagram.

The optical deflection angle of the actuator was tested using a laser vibrometer driven by a steady-state voltage source. The distance from the lens to the screen is denoted as Distance=21 cm, the displacement of the projected light spot center from the coordinate origin is denoted as $X_{s}$, and the formula for calculating the optical angle is $\theta =\arctan(\frac{X_{s}}{\mathrm{Distance}})$. The following shows the relationship graph between the measured voltage U and $X_{s}$ and the calculated optical deflection angle graph，as shown in Figure 14.

Test data shows that the actuator can achieve optical path deflection, outperforming most module-level optical switches

### 3.6. Chapter Summary

The experimental data validate the theoretical predictions. The static voltage–displacement relationship exhibits good linearity, with a sensitivity of S=0.0864 μm/mV. The system’s primary resonance is at 31 Hz. After incorporating the DMR and frame, the out-of-plane displacement at resonance decreased from 57.6 μm to 1.7 μm, and the static Z-direction stiffness increased from 9.62 N/m to 519.3 N/m. Combining the displacement and stiffness data, it is found that the reduction factor for displacement crosstalk differs from that for the force coupling coefficient.

The table highlights the strong consistency between simulation and experiment, with a mere 0.8% error in the primary resonant frequency—directly confirming the reliability of the modal simulation model and the rationality of the synergistic design of the DMR unit and rigid frame. There is no need to measure the moving mass for stiffness derivation; the precise matching of resonant frequencies alone sufficiently supports the core conclusion of “balancing large stroke and high stability.” Notably, the coupled configuration maintains a large stroke of 950.1 μm and dynamic maximum stroke 641.875 μm while achieving low crosstalk and high stability, fully satisfying the stringent requirements of module-level free-space optical interconnect applications, as shown in Table 2.

## 4. Discussion

### 4.1. Physical Mechanism of Displacement Crosstalk Improvement and Analysis of Stiffness–Crosstalk Relationship

Displacement crosstalk (defined as the ratio of the maximum Z-direction displacement to the maximum X-direction displacement) is reduced from 8.97% in the base configuration to 0.265% in the coupled configuration, achieving a 34-fold optimization. This improvement core lies in the synergistic effect of structural stiffness enhancement and magnetic coupling path suppression, with stiffness enhancement serving as the dominant factor for crosstalk suppression. According to basic structural mechanics principles, the out-of-plane displacement Δz and structural stiffness K follow the relationship $\Delta z=\frac{F_{z}}{K}$ (where $F_{z}$ is the Z-direction coupling force), meaning that under the same external force, higher stiffness results in smaller out-of-plane deformation and consequently lower crosstalk. In this design, the synergistic effect of the DMR unit and rigid frame increases the Z-direction stiffness from 9.62 N/m to 519.3 N/m, a 54-fold growth, which directly alters the structural basis for crosstalk generation—in the base configuration, weak Z-direction coupling forces induced by the non-uniform driving magnetic field can cause significant out-of-plane displacement, leading to high crosstalk; in the coupled configuration, the out-of-plane displacement under the same coupling force is reduced to 1/54 of the original, fundamentally eliminating the main source of crosstalk.

The substantial improvement in crosstalk does not rely solely on stiffness enhancement but on the superposition of two effects. Stiffness enhancement directly suppresses out-of-plane deformation through the physical law $\Delta z=\frac{F_{z}}{K}$, which is the primary contributor to crosstalk optimization; at the same time, the symmetric dual-length beam structure of the DMR unit can offset part of the differential magnetic coupling force, and the rigid frame limits the overall torsion of the DMR structure, reducing the coupling path where “X-direction motion induces Z-direction additional displacement”. Both jointly reduce the actual effect of Z-direction coupling forces, further enhancing crosstalk suppression.

Notably, the crosstalk optimization amplitude (34-fold) is slightly lower than the stiffness enhancement amplitude (54-fold), a phenomenon stemming from the complexity of structural–magnetic coupling. Stiffness enhancement is a linear improvement at the pure structural level, following clear mechanical laws; while the suppression of magnetic coupling paths is affected by multiple factors such as magnetic field gradient distribution and structural symmetry, making it impossible to achieve linear optimization fully synchronized with stiffness. This difference precisely indicates that the physical mechanism of crosstalk improvement is not singular but instead is the result of the synergistic effect of structure and magnetic field, and it also explains why traditional designs that only improve stiffness by thickening beams struggle to achieve the same level of crosstalk optimization.

### 4.2. Core Integration Challenges and Proposed Solutions for Module-Level Optical Interconnects

This actuator is targeted at module-level free-space optical interconnect scenarios, which have high requirements for device environmental adaptability and system compactness. Combined with the practical deployment needs of optical interconnect modules and the structural characteristics of the proposed actuator, the following focuses on two core integration challenges and the engineering solutions we plan to implement:

Challenge of thermal stability over a wide temperature range: Industrial operating temperatures of optical interconnect modules typically range from −40~80 °C. Differences in the coefficient of thermal expansion (CTE) between silicon-based structures and permanent magnets can easily cause out-of-plane displacement drift under long-term temperature fluctuations, affecting optical coupling stability. To address this issue, we plan to adopt a combined strategy of “passive thermal matching + active algorithm compensation”. At the passive level, we intend to select a material combination with small CTE differences—silicon has a CTE of approximately $2.6\times 10^{-6}$/°C, NdFeB permanent magnet around $3.1\times 10^{-6}$/°C, and the epoxy adhesive for bonding will prioritize a type with CTE $5.0\times 10^{-6}$/°C. This combination is widely used in silicon-based MEMS optical devices, which can provide a basic guarantee for thermal stability. At the active level, we plan to integrate a micro-temperature sensor in the module to collect ambient temperature in real time, and, then, dynamically adjust the driving voltage to offset drift based on the temperature–displacement relationship model to be calibrated through subsequent experiments. Current preliminary tests show that the displacement drift per 10 °C can be controlled within 0.3 μm only through material matching, and it is expected to be further reduced to within 0.1 μm after combining with algorithm compensation, meeting the stable operation requirements of optical interconnect modules.

Challenge of drive circuit integration: Module-level systems have strict control over overall volume. Discrete drive circuits will increase packaging complexity and may introduce additional signal interference. For this reason, we plan to design a miniaturized PCB compatible with the actuator’s dimensions—the preliminary planned size is 15 mm × 15 mm, which perfectly matches the actuator’s 13 mm × 13 mm main structure. This PCB is intended to integrate the five-turn planar spiral coil, current amplifier (designed gain 100×), and feedback sensor interface required by the actuator. Compared with traditional discrete circuits, it is expected to reduce the drive system volume by about 40%. At the same time, we plan to optimize the circuit layout, controlling the lead length between the coil and the drive chip within 5 mm, so as to reduce the interference of parasitic inductance on the drive signal and improve the dynamic response stability of the actuator. This miniaturized integration scheme refers to the mature circuit design ideas of existing MEMS electromagnetic actuators, with high engineering feasibility. Subsequent work will include PCB prototyping and performance verification.

## Figures and Tables

**Table 1:** Parameters and their definitions.

Parameter	Description
$L_{1}$	Folded-beam segment length (segment 1)
$L_{2}$	Folded-beam segment length (segment 2)
W	Beam width
T	Beam thickness
$z_{0}$	Analysis height above coil
$L_{d1}$	DMR structural beam 1 length
$L_{d2}$	DMR structural beam 2 length
$W_{d}$	Width of DMR structural beams
R	The equivalent arm of the DMR structure to the center of gravity
$L_{f}$	Beam length of square frame
Distance	Distance from lens to screen
$X_{s}$	Displacement from the center of transmitted light to the origin of coordinates
θ	Light deflection angle
E	Young’s modulus
I	The section moment of inertia

**Table 2:** Comparison of performance index experimental measurement and simulation results.

Performance Indicator	Simulation Result	Experimental Result
Maximum X-direction displacement	950 μm	950.1 μm
Primary resonant frequency	31.26 Hz	31 Hz
Displacement crosstalk	Significantly suppressed	0.265%
Light deflection angle ( θ)	-	±21°

## Data Availability

Data will be made available on request.

## Abbreviations

The following abbreviations are used in this manuscript:

DMR	Differential Motion Rejection
